# Endovascular Treatment of Ruptured and Unruptured Wide-Neck Intracranial Aneurysms Using LVIS EVO Stent-Assisted Coiling: Mid-Term Results

**DOI:** 10.3390/jcm14238457

**Published:** 2025-11-28

**Authors:** Wojciech Poncyljusz, Kinga Kubiak, Elżbieta Włodarczyk, Konrad Jarosz, Leszek Sagan

**Affiliations:** 1 Department of Diagnostic Imaging and Interventional Radiology, Pomeranian Medical University, Rybacka 1, 70-204 Szczecin, Poland; 2 Department of Neurosurgery and Pediatric Neurosurgery, Pomeranian Medical University, Rybacka 1, 70-204 Szczecin, Poland; 3 Anesthesiology and Intensive Care Department, Pomeranian Medical University, Rybacka 1, 70-204 Szczecin, Poland

**Keywords:** intracranial aneurysm, embolization, LVIS EVO stent

## Abstract

**Background**: Stent-assisted coiling (SAC) is an established treatment for wide-neck intracranial aneurysms. The LVIS EVO stent is a new-generation braided device with improved navigability and radiopacity. We evaluated the safety, feasibility, and mid-term outcomes of LVIS EVO SAC in ruptured and unruptured aneurysms. **Methods**: We retrospectively analyzed 242 consecutive patients treated and evaluated from 2020 to 2025; 63 (26.0%) presented with ruptured and 179 (74.0%) with unruptured aneurysms. Aneurysm occlusion was graded by the Raymond–Roy occlusion classification (RROC) on immediate DSA and at 12–18 months using 3T MR angiography. Clinical outcomes were assessed with the modified Rankin Scale (mRS). **Results**: Stent delivery succeeded in all cases. Adjunctive angioplasty was required in three procedures. Thromboembolic events occurred in six patients, including four in-stent thromboses treated with eptyfibatide, procedure-related SAH in three, and femoral hematomas in five. Mid-term imaging was available in 228 patients (51 SAH, 177 non-SAH). Adequate occlusion (RROC I–II) was achieved in 48/51 SAH (94.1%) and 169/177 non-SAH (95.5%). Residual sac filling (RROC III) occurred in 11/228 (4.8%), and all retreated. Favorable outcome (mRS 0–2) was observed in 49/63 SAH (77.8%) and 170/179 non-SAH (95.0%). Mortality was 12/63 (19.0%) in SAH—attributed to initial hemorrhage severity—and 2/179 (1.1%) in non-SAH (Takotsubo syndrome, leukemia). **Conclusions**: LVIS EVO SAC is a safe and effective option for ruptured and unruptured wide-neck aneurysms, yielding high rates of durable occlusion and favorable functional outcomes. Mid-term data support its reliability as an alternative to other endovascular strategies.

## 1. Introduction

Endovascular therapy of intracranial aneurysms has become increasingly widespread, offering lower morbidity and mortality compared with surgical clipping. However, treatment of wide-neck and complex aneurysms remains technically challenging. Stand-alone coil embolization is feasible mainly in aneurysms with a favorable dome-to-neck ratio value, while wide-neck aneurysms frequently require adjunctive techniques [1].

Since the first description of stent-assisted coiling (SAC) in 1997, intracranial stents have been shown to provide adequate mechanical support for coil placement, improve long-term occlusion stability, and induce favorable hemodynamic changes within the aneurysm sac. Several devices have been developed since then, including laser-cut stents such as Enterprise stent (Codman Neuro, Raynham, MA, USA) and Neuroform stent (Stryker Neurovascular, Fremont, CA, USA) [2,3], and braided stents such as LVIS and LVIS Jr (MicroVention-Terumo, Aliso Viejo, CA, USA) [4,5,6]. These advances have expanded the scope of endovascular treatment, but challenges remain in achieving durable occlusion in wide-neck bifurcation aneurysms, especially in ruptured cases.

The LVIS EVO (MicroVention-Terumo, Aliso Viejo, CA, USA) is the most recent generation of self-expandable braided stents. It is manufactured using drawn-filled tube (DFT) technology, which improves radiopacity under fluoroscopy, and incorporates shorter flared ends to facilitate deployment in tortuous anatomy [7,8]. Compared with its predecessors, EVO offers a smaller cell size and greater conformability, theoretically improving coil stability and vessel wall apposition [8].

Alternative strategies for wide-neck aneurysms include flow-diverter devices such as Pipeline (Medtronic, Minneapolis, MN, USA), FRED (MicroVention-Terumo, Aliso Viejo, CA, USA), Surpass (Stryker Neurovascular, Fremont, CA, USA), and others. These types of solutions have demonstrated high long-term occlusion rates in large or giant sidewall aneurysms [9,10,11,12,13], but their use in bifurcation and ruptured aneurysms remains limited due to the need for dual antiplatelet therapy and concerns about delayed ischemic or hemorrhagic complications [14,15]. Another approach involves intrasaccular flow-disruption devices, such as the WEB (MicroVention-Terumo, Aliso Viejo, CA, USA) system, Luna/Artisse/Medina (Medtronic, Irvine, CA, USA), and Contour (Cerus Endovascular, Fremont, CA, USA). WEB has shown promising results in wide-neck bifurcation aneurysms, including ruptured cases; however, complete occlusion rates remain lower than those achieved with SAC, and their long-term durability is still debated [16,17,18].

Despite these alternatives, clinical data specifically addressing the LVIS EVO remain limited. Only small series or multicenter feasibility reports have been published until today [1,7,8,19,20], and evidence regarding its use in both ruptured and unruptured aneurysms in a large, consecutive cohort is scarce.

The aim of the present study was therefore to evaluate the safety, feasibility, and mid-term outcomes of LVIS EVO stent-assisted coiling in a consecutive series of patients with ruptured and unruptured intracranial aneurysms. The present study was specifically designed to address these gaps by providing one of the largest single-center cohorts treated consecutively with LVIS EVO, including both ruptured and unruptured wide-neck aneurysms, and using standardized 3T MR angiographic follow-up at 12–18 months to ensure consistent evaluation of treatment stability.

## 2. Materials and Methods

### 2.1. Study Design and Patient Population

This single-center observational study included consecutive patients who underwent LVIS EVO stent-assisted coiling between February 2020 and August 2025. This was a retrospective analysis of a prospectively maintained institutional database. All clinical, procedural, and follow-up data were collected prospectively as part of routine clinical practice. For the purpose of this study, the dataset was reviewed retrospectively. No relevant variables were missing; complete datasets were available for all, including patients. Eligible cases comprised wide-neck aneurysms, ruptured or unruptured. Rupture was defined as presentation with acute subarachnoid hemorrhage (SAH) confirmed by computed tomography (CT) or lumbar puncture. Unruptured aneurysms were identified incidentally or in association with chronic symptoms. Patients with fusiform, dissecting, or blister-like aneurysms were excluded.

### 2.2. Ethical Approval and Consent

The study protocol was approved by the Bioethics Committee (approval code: KB-0012/29/05/2020/Z, approval date: 21 May 2020). Written informed consent was obtained from all patients or their legal representatives prior to treatment, in accordance with the Declaration of Helsinki.

### 2.3. Endovascular Procedure

All procedures were performed under general anesthesia with systemic heparinization. A transfemoral approach with a 6F introducer sheath (Cordis, Bridgewater, NJ, USA) was used in all cases. Depending on the location of the aneurysm, a Chaperon 6F guiding catheter (MicroVention-Terumo, Aliso Viejo, CA, USA) was positioned in the internal carotid or vertebral artery. Standard digital subtraction angiography (DSA) and three-dimensional rotational angiography were performed to evaluate aneurysm morphology and select the optimal working projection.

**Figure 1:**
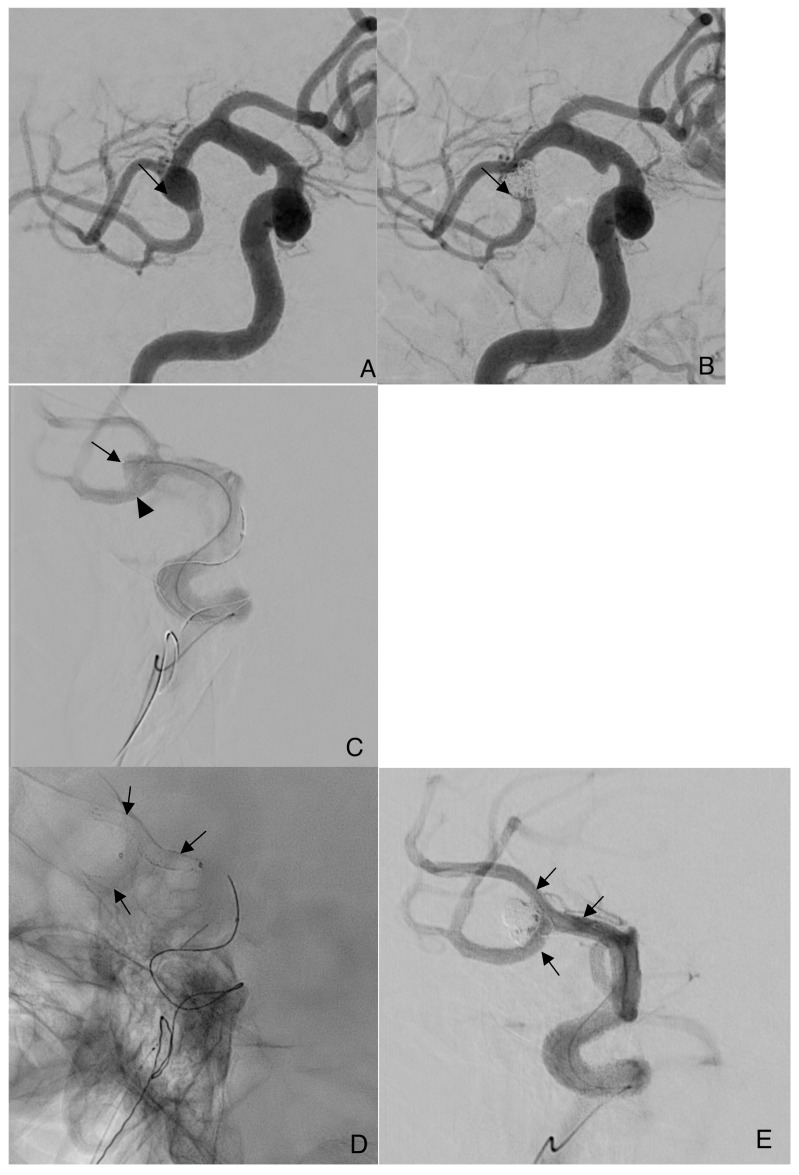
Representative case of LVIS EVO stent-assisted coiling of a wide-neck MCA bifurcation aneurysm. (**A**,**B**) Pre- and post-treatment DSA in oblique 30° projection, showing complete exclusion of the aneurysm sac after LVIS EVO stent-assisted coiling (arrows). (**C**) Jailing technique: fluoroscopic image with the microcatheter positioned inside the aneurysm sac (arrow) after deployment of the first LVIS EVO stent (arrowhead), prior to coil placement. (**D**,**E**) Fluoroscopic views of Y-stent configuration (arrows) with two LVIS EVO stents deployed at the bifurcation. The stents are clearly visualized due to their radiopacity. Final embolization in the working projection.

The LVIS EVO stent was delivered through a Headway 17 microcatheter. The microcatheter was positioned over a Traxcess 0.014-inch guidewire (both MicroVention, Aliso Viejo, CA, USA). The jailing technique Headway Duo microcatheter is used in all patients for coil delivery; in one case, a Y-stent configuration is required (Figure 1). Vaso-CT (Philips Clarity DSA system) was performed in all patients to confirm stent apposition to the artery wall. In cases of incomplete stent expansion, adjunctive in-stent angioplasty was performed. In selected bifurcation aneurysms, the EVO stent was deliberately molded by advancing the struts toward the aneurysm neck to optimize coverage. This maneuver, feasible due to the high conformability of the device, allowed secure protection of the aneurysm neck, a technique not possible with laser-cut stents.

### 2.4. Antiplatelet Therapy

For unruptured aneurysms, dual antiplatelet therapy (acetylsalicylic acid 150 mg and clopidogrel 75 mg daily) was administered for at least seven days prior to the procedure. In the SAH cohort, loading doses of ticagrelor (180 mg) plus acetylsalicylic acid (300 mg) were administered one hour before the intervention. All patients continued dual antiplatelet therapy for a minimum of 3 months after treatment, followed by acetylsalicylic acid monotherapy for 6 months.

### 2.5. Follow-Up Protocol

Periprocedural flat-panel CT (Vaso-CT) was performed in all patients to evaluate stent deployment and wall apposition. Immediate angiographic outcome was classified according to the Raymond–Roy Occlusion Classification (RROC) on DSA at the end of the procedure. Follow-up imaging was systematically performed between 12 and 18 months using 3T contrast-enhanced MR and MR angiography (Signa Pioneer, GE Healthcare, Chicago, IL, USA). This protocol, previously extensively validated at our center, allows reliable detection of even small neck remnants (1–2 mm), which cannot be consistently assessed with 1.5T MR. Importantly, the EVO stent produces minimal artifacts on MR, facilitating follow-up evaluation compared with some flow-diverter devices. Clinical outcomes were assessed using the modified Rankin Scale (mRS) at discharge and at the 3-month follow-up visit. The 12–18-month interval was chosen in accordance with the current endovascular literature defining mid-term outcomes, as the majority of recanalization or residual filling events after SAC occur within the first postoperative year, with limited additional changes beyond 18 months.

### 2.6. Retreatment Policy

Per protocol, all patients with residual aneurysm filling (RROC III) on 12–18-month MR angiography underwent additional embolization. The retreatment rate was calculated as the proportion of patients requiring such re-intervention during follow-up.

### 2.7. Study Endpoints

- Primary angiographic endpoints:

○ Complete aneurysm occlusion (RROC I–II) on post-procedural DSA.
○ Adequate occlusion (RROC I–II) on 12–18-month follow-up MR with Gd-DTPA and MR-angiography.

- Secondary angiographic endpoints:

○ Complete aneurysm occlusion (RROC I).
○ Recurrence or residual filling.
○ Retreatment.

- Clinical endpoints:

○ Favorable functional outcome (mRS 0–2).
○ Any periprocedural and postprocedural complications (ischemic or hemorrhagic).
○ Periprocedural morbidity and mortality.
○ All-cause mortality during follow-up.

### 2.8. Statistical Analysis

Baseline characteristics, procedural details, and outcomes were summarized separately for the SAH and no-SAH subgroups. Continuous variables were reported as mean ± SD or median (IQR) as appropriate and were compared using Student’s *t*-test or the Mann–Whitney U test, as appropriate, based on normality assessed with the Shapiro–Wilk test. Categorical variables were compared using the χ^2^ test or Fisher’s exact test. A two-sided *p* < 0.05 was considered statistically significant. All analyses were performed using standard statistical software STATISTICA (version 13.3; TIBCO Software Inc., Palo Alto, CA, USA).

## 3. Results

### 3.1. Patient Population

A total of 242 patients (188 women, 54 men; mean age 59.6 ± 13.6 years) were treated with LVIS EVO stent-assisted coiling between February 2020 and August 2025. Of these, 63 (26.0%) presented with ruptured (SAH) and 179 (74.0%) had unruptured (no-SAH) aneurysms. The patients’ characteristics and aneurysm location are summarized in Table 1. Patients with SAH were younger than those without SAH (56.0 ± 12.4 vs. 60.9 ± 13.8 years; *p* = 0.012). Female patients were less common in the ruptured cohort. (61.8%) compared with the unruptured cohort (80.5%; *p* = 0.004). Mean aneurysm maximum diameter was 6.7 ± 3.5 mm (SAH) and 7.3 ± 3.9 mm (no-SAH; *p* = 0.21). Mean neck width was 3.3 ± 1.1 mm vs. 3.7 ± 1.3 mm (*p* = 0.18), resulting in dome-to-neck ratios approximating 2.0 in both groups.

Given that the anterior communicating artery (AComA) was the most frequent aneurysm location in our series (24/63 in SAH and 30/179 in non-SAH), a focused sub-analysis was performed. Complete occlusion (RROC I) was achieved in 45/54 (83.3%) AComA aneurysms, with adequate occlusion (RROC I–II) in 52/54 (96.3%) cases at 12–18-month follow-up. Adjunctive angioplasty was required in only one patient (1.9%), and no in-stent thrombosis or delayed rebleeding was observed. All patients with unruptured AComA aneurysms achieved a favorable clinical outcome (mRS 0–2), whereas 79.2% of ruptured AComA aneurysms reached the same endpoint. These results underscore the technical versatility of LVIS EVO in this anatomically challenging location.

### 3.2. Procedural Details and Angiographic Outcomes

The procedural details are presented in Table 2. All aneurysms were treated with LVIS EVO stent-assisted coiling using the jailing technique. Stent delivery to the intended location was successful in all cases. Incomplete wall apposition requiring adjunctive in-stent angioplasty occurred in 3 patients (1.2%). One patient in the SAH cohort required a Y-stent configuration. Vascular closure was performed with Femoseal (Terumo Corporation, Tokyo, Japan) in 190 cases (78.5%) and Angioseal (Terumo Corporation, Tokyo, Japan) in 52 cases (21.5%) (Terumo, Tokyo, Japan). Immediate post-procedural DSA demonstrated Raymond–Roy class I or II occlusion in the majority of patients. At mid-term follow-up (12–18 months), imaging was available in 228 patients (51 SAH, 177 non-SAH). Adequate occlusion (RROC I–II) was achieved in 48/51 SAH (94.1%) and 169/177 unruptured aneurysms (95.5%; *p* = 0.62). Complete occlusion (RROC I) was achieved in 44/51 (86.3%) SAH and 136/177 (76.8%) unruptured aneurysms. Residual filling (RROC III) occurred in 11 patients (4.8%) overall, similar in both groups: SAH: 3/51 (5.9%), no-SAH: 8/177 (4.5%), all requiring retreatment after MR 12–18 months control.

### 3.3. Clinical Outcomes

The median mRS at discharge was two in the ruptured cohort and one in the unruptured cohort. At 12–18 months, a favorable outcome (mRS 0–2) was achieved by 197 patients (86.4%), including 77.8% patients with ruptured aneurysms and 95.0% of unruptured. Poor outcome (mRS 3–5) was observed in 31 patients (13.6%). Overall mortality was 14 patients (5.8%): 12/63 (19.0%) in the SAH group and 2/179 (1.1%) in the unruptured group (*p* < 0.001). Among patients with unruptured aneurysms, deaths occurred but were not attributed to the procedure (Takotsubo syndrome, *n* = 1; leukemia, *n* = 1), whereas all deaths in the SAH group reflected the severity of the initial hemorrhage and poor baseline mRS grade, without deterioration attributable to the intervention. Table 3 summarizes the angiographic and clinical outcomes.

### 3.4. Complications and Mortality

Periprocedural complications were observed in a minority of cases. Thromboembolic events occurred in 6 patients (2.5%). In-stent thrombosis was noted in 4 patients (1.6%), all treated with eptifibatide. Intraoperative rupture was observed in 3 patients (1.2%). Procedure-related SAH occurred in 3 patients (1.2%). Femoral access-site hematomas were reported in 5 patients (2.1%); none required surgical intervention. No delayed aneurysm rupture was observed during follow-up. Among patients with subarachnoid hemorrhage, 28.6% presented with Hunt–Hess grade ≥3 and 69.8% Fisher grade 3–4 bleeding, explaining the 19% mortality observed in this subgroup, which aligns with prior reports of high-grade SAH outcomes (15–25% mortality) and supports the conclusion that deaths were primarily due to the initial hemorrhage severity rather than procedural complications.

## 4. Discussion

### 4.1. Key Findings

In this large single-center cohort, LVIS EVO stent-assisted coiling demonstrated feasibility and safety in both ruptured and unruptured wide-neck aneurysms. Immediate angiographic results demonstrated a high rate of adequate occlusion, and follow-up imaging at 12–18 months confirmed durable stability. Importantly, complete occlusion was achieved in over 80% of cases, and adequate occlusion (RROC I–II) was 94.1% in SAH and 95.5% in unruptured aneurysms, with residual filling (RROC III) in 11 patients (4.8%), all requiring retreatment. Functional outcomes were favorable in most patients, particularly in the unruptured cohort (mRS 0–2 in 95%). Periprocedural complication rates were low and included thromboembolic events, in-stent thrombosis managed with Integrilin (with one unsuccessful thrombectomy attempt), procedure-related SAH, and femoral access-site hematomas. Mortality was 19.0% in SAH patients (all due to initial hemorrhage severity) and 1.1% in unruptured patients (due to Takotsubo syndrome and leukemia). These results highlight LVIS EVO as a reliable treatment option for complex aneurysms, including those presenting with SAH.

### 4.2. Comparison with Prior EVO Series

Our outcomes are in line with some previously published experiences with the LVIS EVO device. Maurer et al. [1] reported complete occlusion in 79% at 6 months and 82% at 12–18 months, less but comparable to our series. In an observational multicenter study, Vollherbst [7] reported a complete occlusion rate in 54% (Class I), but the most frequent aneurysm locations were the middle cerebral artery (25.4%), and the data were collected from the initial phase of introducing the LVIS EVO stent in the centers, which could have been reflected in the statistics. Kayan in multicenter early United States reported that Class I immediate occlusion was obtained in 33%, Class II in 22%, Class IIIa in 37% and Class IIIb in 8% of cases [19]. These outcomes were inferior and not directly comparable with those of coiling alone, likely reflecting several contributing factors: 16 centers participated in the study, with an average of 3.3 stents implanted per center, and coils were placed in 48 cases (87.2%). Our results are most consistent with the published results by Mosimann. Complete occlusion in this series was 97% initially and 90% at 12 months (103 aneurysms) [20]. Our findings strengthen these results by providing one of the largest single-center analyses, including both ruptured and unruptured aneurysms with systematic MR follow-up.

### 4.3. Comparison with Other Stents

Earlier generation laser-cut stents, such as Enterprise and Neuroform, improved the feasibility of SAC but showed limitations in flexibility and vessel wall apposition [2,3,21]. Braided devices such as LVIS and LVIS Jr offered better scaffolding and lower recurrence rates [4,5,6]. EVO combines these advantages with enhanced fluoroscopic visibility and smaller cell size [1,7,8,19,20]. In our experience, EVO deployment was successful in all cases, with adjunctive angioplasty required in only 1.2%, underscoring its technical reliability. Importantly, due to its conformability, the EVO stent could be intentionally molded at bifurcation necks to achieve more complete neck coverage by gently pressing the struts against the aneurysm neck—an approach not feasible with laser-cut stents. This technique, used selectively for bifurcation aneurysms in our cohort, requires substantial operator experience and deliberate stent manipulation; beyond straightforward deployment, controlled stent shaping may influence treatment durability. Other braided stents, such as Leo/Leo Baby (Balt Extrusion, Montmorency, France) and Accero (Acandis GmbH, Pforzheim, Germany), have also shown high technical success and durable aneurysm occlusion, particularly in small-vessel or distal anatomies, but direct comparative data with EVO remain limited [21,22,23].

In recent years, stents and flow diverters with antithrombogenic surface coatings have been introduced, including the HEAL technology (Accero Heal and Derivo 2 Heal) and the hydrophilic polymer coating (HPC) used in p48/p64 MW flow diverters and the pEGASUS-HPC stent system [24,25,26]. Early clinical reports suggest that these coatings reduce device thrombogenicity and may allow treatment under prasugrel single antiplatelet therapy, while achieving aneurysm occlusion rates comparable to conventional bare-metal devices. However, current evidence is based on small, retrospective series with short follow-up, and no head-to-head comparisons with bare-metal braided stents such as LVIS EVO exist. Our results should therefore be interpreted as representative of standard DAPT-based, bare-metal stent–assisted coiling.

### 4.4. Comparison with WEB and Other Intrasaccular Devices

Intrasaccular flow-disruption devices such as the WEB system and Luna/Artisse (Medtronic) have emerged as alternatives for bifurcation aneurysms. The CLEVER trial [17] demonstrated the safety and feasibility of WEB in both ruptured and unruptured cases, but complete occlusion rates (~80%) remain lower than those achieved with the present study with LVIS EVO. Similar limitations were observed with Luna/Artisse [18]. In an experienced center, LVIS EVO can provide higher rates of durable occlusion, particularly in ruptured aneurysms, suggesting an advantage over intrasaccular devices in selected anatomies.

### 4.5. Comparison with Flow Diversion

Flow-diverter stents such as Pipeline, FRED, and Surpass have revolutionized the treatment of large and giant sidewall aneurysms, with long-term occlusion rates >90% [10,11,12,13,14,15]. However, their use in ruptured aneurysms and bifurcation anatomy is limited by the need for dual antiplatelet therapy anyway and the risk of delayed ischemic or hemorrhagic complications [10,11,12,13,14,15]. Other devices, such as Silk Vista Baby (Balt Extrusion, Montmorency, France), p64 and p48 (Phenox GmbH, Bochum, Germany), Derivo (Acandis GmbH, Pforzheim, Germany), and Tubridge (MicroPort NeuroTech, Shanghai, China) have also demonstrated high efficacy in unruptured aneurysms, though their use in bifurcation and ruptured aneurysms remains restricted for the same reasons [27,28,29,30]. Our data confirms that EVO can be safely used in both acute SAH and elective settings, providing a complementary option where flow diversion is contraindicated.

From an economic standpoint, the use of the LVIS EVO stent combined with detachable coils represents a substantially less expensive strategy than intrasaccular devices such as the WEB system. In our institution, the total device cost of a single WEB procedure is approximately twice that of one LVIS EVO stent plus coils, while a flow-diverter procedure is roughly 30% more expensive. Moreover, published series have reported retreatment rates of 8–12% after WEB implantation [16,17,18], whereas in our cohort, only 4.8% of patients required re-embolization after LVIS EVO SAC. Considering both procedural expenses and durability, LVIS EVO may therefore provide a cost-effective and resource-efficient option, particularly in healthcare systems with constrained budgets.

### 4.6. Strengths and Limitations

The strengths of this study include a relatively large cohort (*n* = 242), systematic follow-up at 12–18 months, and separate analysis of ruptured and unruptured aneurysms. Another strength is the use of a standardized 3T contrast-enhanced MR angiography protocol, which in our center has become a reliable method for detecting small neck remnants (1–2 mm). Importantly, EVO generates minimal MR artifacts, facilitating non-invasive follow-up compared with some flow-diverter devices. Limitations include the single-center design, reliance on MR rather than DSA for follow-up, and lack of randomization. In addition, long-term durability beyond 18 months could not yet be assessed. We are currently initiating a prospective multicenter registry evaluating LVIS EVO outcomes in both ruptured and unruptured aneurysms with extended follow-up beyond 5 years.

### 4.7. Clinical Implications and Future Directions

Our findings support the use of LVIS EVO as a versatile and effective option for wide-neck intracranial aneurysms, including selected ruptured lesions in which alternatives (e.g., WEB or flow diversion) may be less suitable. Prospective, multicenter studies with extended follow-up are warranted to confirm these results and to delineate the device’s optimal role within contemporary endovascular practice. Given its favorable performance, procedural reliability, and approximately two-fold lower device cost compared with the WEB system and 30% lower cost compared with flow-diverters, LVIS EVO may represent a practical and cost-effective endovascular strategy across a broad spectrum of aneurysm morphologies.

## Figures and Tables

**Table 1:** Baseline characteristics, comorbidities, and aneurysm location.

Characteristic	SAH (*n* = 63)	No-SAH (*n* = 179)	*p*-Value
Age, mean ± SD (years)	56.0 ± 12.4	60.9 ± 13.8	0.012
Female sex, *n* (%)	39 (61.8%)	144 (80.5%)	0.004
Hypertension, *n* (%)	40 (63.5%)	112 (62.6%)	0.91
Diabetes mellitus, *n* (%)	6 (9.5%)	17 (9.5%)	0.99
Smoking, *n* (%)	22 (34.9%)	51 (28.5%)	0.35
Multiple aneurysms, *n* (%)	7 (11.1%)	26 (14.5%)	0.53
Coronary artery disease, *n* (%)	8 (12.7%)	21 (11.7%)	–
Atrial fibrillation, *n* (%)	3 (4.8%)	7 (3.9%)	–
Hyperlipidemia, *n* (%)	12 (19.0%)	36 (20.1%)	–
Aneurysm location			
AComA	24 (38.1%)	30 (16.8%)	
ICA	23 (36.5%)	87 (48.6%)	
MCA	9 (14.3%)	34 (19.0%)	
ACA	0	4 (2.2%)	
BA	5 (7.9%)	15 (8.4%)	
VA	0	4 (2.2%)	
PComA	2 (3.2%)	3 (1.7%)	
PICA	0	2 (1.1%)	

**Table 2:** Procedural details.

Parameter	SAH (*n* = 63)	No-SAH (*n* = 179)	Total (*n* = 242)
Stent delivery success	63 (100%)	179 (100%)	242 (100%)
Adjunctive in-stent angioplasty	1 (1.6%)	2 (1.1%)	3 (1.2%)
Y-stent configuration	0	1 (0.6%)	1 (0.4%)
Thromboembolic events	2 (3.2%)	4 (2.2%)	6 (2.5%)
In-stent thrombosis	1 (1.6%)	3 (1.7%)	4 (1.7%)
Intraoperative rupture	2 (3.2%)	1 (0.6%)	3 (1.2%)
Procedure-related SAH	2 (3.2%)	1 (0.6%)	3 (1.2%)
Femoral hematoma (conservative)	2 (3.2%)	3 (1.7%)	5 (2.1%)
Closure device Femoseal	50 (79.4%)	140 (78.2%)	190 (78.5%)
Closure device Angioseal	13 (20.6%)	39 (21.8%)	52 (21.5%)

**Table 3:** Angiographic and clinical outcomes.

Outcome	SAH (*n* = 63)	No-SAH (*n* = 179)	Total (*n* = 242)
Follow-up available	51	177	228
Adequate occlusion (RROC I–II)	48/51 (94.1%)	169/177 (95.5%)	217/228 (95.2%)
Complete occlusion (RROC I)	42/51 (82.4%)	146/177 (82.5%)	188/228 (82.5%)
Residual filling (RROC III)	3/51 (5.9%)	8/177 (4.5%)	11/228 (4.8%)
Retreatment (12–18 months)	3 (4.8%)	8 (4.5%)	11 (4.8%)
Favorable outcome (mRS 0–2)	49 (77.8%)	170 (95.0%)	219 (90.5%)
Mortality	12 (19.0%)	2 (1.1%)	14 (5.8%)

## Data Availability

The original contributions presented in this study are included in the article. Further inquiries can be directed to the corresponding author.

## Abbreviations

The following abbreviations are used in this manuscript:

SAC	Stent-assisted coiling
RROC	Raymond-Roy Occlusion Classification
SAH	Subarachnoid hemorrhage
DSA	Digital subtraction angiography

## References

[1] Maurer C.J., Berlis A., Maus V., Behrens L., Weber W., Fischer S. (2023). Treatment of broad-based intracranial aneurysms with the LVIS EVO stent: A retrospective observational study at two centers with short- and mid-term follow-up. Sci. Rep..

[2] Jankowitz B.T., Hanel R., Jadhav A.P., Loy D.N., Frei D., Siddiqui A.H., Puri A.S., Khaldi A., Turk A.S., Malek A.M. (2019). Neuroform Atlas Stent System for the treatment of intracranial aneurysm: Primary results of the ATLAS Humanitarian Device Exemption cohort. J. Neurointerv. Surg..

[3] Kadkhodayan Y., Rhodes N., Blackburn S., Derdeyn C.P., Cross D.T., Moran C.J. (2013). Comparison of Enterprise with Neuroform stent-assisted coiling of intracranial aneurysms. AJR Am. J. Roentgenol..

[4] Fiorella D., Boulos A., Turk A.S., Siddiqui A.H., Arthur A.S., Diaz O., Lopes D.K. (2019). The safety and effectiveness of the LVIS stent system for the treatment of wide-necked cerebral aneurysms: Final results of the pivotal US LVIS trial. J. Neurointerv. Surg..

[5] Forestier G., Piotin M., Chau Y., Derelle A.-L., Brunel H., Aggour M., Saleme S., Levrier O., Pierot L., Barreau X. (2024). Safety and effectiveness of the LVIS and LVIS Jr devices for the treatment of intracranial aneurysms: Final results of the LEPI multicenter cohort study. J. Neuroradiol..

[6] Poncyljusz W., Biliński P., Safranow K., Baron J., Zbroszczyk M., Jaworski M., Bereza S., Burke T.H. (2015). The LVIS/LVIS Jr. stents in the treatment of wide-neck intracranial aneurysms: Multicentre registry. J. Neurointerv. Surg..

[7] Vollherbst D.F., Berlis A., Maurer C., Behrens L., Sirakov S., Sirakov A., Fischer S., Maus V., Holtmannspötter M., Rautio R. (2021). Periprocedural Safety and Feasibility of the New LVIS EVO Device for Stent-Assisted Coiling of Intracranial Aneurysms: An Observational Multicenter Study. AJNR Am. J. Neuroradiol..

[8] Poncyljusz W., Kubiak K. (2020). Initial experience with LVIS EVO stents for the treatment of intracranial aneurysms. J. Clin. Med..

[9] Becske T., Brinjikji W., Potts M.B., Kallmes D.F., Shapiro M., Moran C.J., Levy E.I., McDougall C.G., Szikora I., Lanzino G. (2017). Long-Term Clinical and Angiographic Outcomes Following Pipeline Embolization Device Treatment of Complex Internal Carotid Artery Aneurysms: Five-Year Results of the Pipeline for Uncoilable or Failed Aneurysms Trial. Neurosurgery.

[10] McDougall C.G., Diaz O., Boulos A., Siddiqui A.H., Caplan J., Fifi J.T., Turk A.S., Kayan Y., Jabbour P., Kim L.J. (2022). Safety and efficacy results of the Flow Redirection Endoluminal Device (FRED) stent system in the treatment of intracranial aneurysms: US pivotal trial. J. Neurointerv. Surg..

[11] Meyers P.M., Coon A.L., Kan P.T., Wakhloo A.K., Hanel R.A. (2019). SCENT Trial. Stroke.

[12] Wakhloo A.K., Lylyk P., de Vries J., Taschner C., Lundquist J., Biondi A. (2015). Surpass flow diverter in the treatment of intracranial aneurysms: A prospective multicenter study. AJNR Am. J. Neuroradiol..

[13] Kallmes D.F., Brinjikji W., Cekirge S., Fiorella D., Hanel R.A., Jabbour P., Lopes D., Lylyk P., McDougall C.G., Siddiqui A. (2017). Safety and efficacy of the Pipeline Embolization Device for treatment of intracranial aneurysms: A pooled analysis of 3 large studies. J. Neurosurg..

[14] Zhou G., Su M., Yin Y.L., Li M.-H. (2017). Complications associated with the use of flow-diverting devices for cerebral aneurysms: A systematic review and meta-analysis. Neurosurg. Focus.

[15] Brinjikji W., Murad M.H., Lanzino G., Cloft H.J., Kallmes D.F. (2013). Endovascular treatment of intracranial aneurysms with flow diverters: A meta-analysis. Stroke.

[16] Pierot L., Moret J., Barreau X., Szikora I., Herbreteau D., Turjman F., Holtmannspötter M., Januel A.-C., Costalat V., Fiehler J. (2017). Safety and efficacy of aneurysm treatment with WEB in the cumulative population of three prospective, multicenter series. J. Neurointerv. Surg..

[17] Spelle L., Costalat V., Caroff J., Wodarg F., Fischer S., Herbreteau D., A Möhlenbruch M., Januel A.-C., Papagiannaki C., Klisch J. (2024). CLinical EValuation of WEB 17 device in intracranial aneuRysms (CLEVER): Procedural, 30-day and 1-year safety results for ruptured and unruptured aneurysms. J. Neurointerv. Surg..

[18] Hecker C., Broussalis E., Griessenauer C.J., Killer-Oberpfalzer M. (2023). A mini-review of intrasaccular flow diverters. J. Neurointerv. Surg..

[19] Kayan Y., Delgado Almandoz J.E., Copelan A., Matouk C., Chaudry M.I., Altschul D., Essibayi M.A., Goren O., Yim B., Tsappidi S. (2025). Multicenter early United States feasibility study and periprocedural safety of LVIS EVO for the treatment of unruptured intracranial aneurysms. J. Neurointerv. Surg..

[20] Mosimann P.J., Yamac E., Wallocha M., Ayad A., Chapot R. (2024). LVIS EVO stent-through-balloon after hydrocoil embolization of intracranial aneurysms: One-year results. Interv. Neuroradiol..

[21] Cagnazzo F., Cappucci M., Lefevre P.H., Dargazanli C., Gascou G., Morganti R., Mazzotti V., di Carlo D., Perrini P., Mantilla D. (2018). Treatment of Intracranial Aneurysms with Self-Expandable Braided Stents: A Systematic Review and Meta-Analysis. AJNR Am. J. Neuroradiol..

[22] Tang H., Lu Z., Zeng Z., Li S., Shang C., Zuo Q., Liu J., Huang Q. (2024). Treatment of saccular wide-neck intracranial aneurysm using Leo baby stent: A single-center experience based on 156 cases. Neurosurg. Rev..

[23] Nania A., Dobbs N., DuPlessis J., Keston P., Downer J. (2021). Early experience treating intracranial aneurysms using Accero: A novel, fully visible, low profile braided stent with platinum-nitinol composite wire technology. J. Neurointerv. Surg..

[24] Hellstern V., Aguilar Perez M., Henkes E., Donauer E., Wendl C., Bäzner H., Henkes H. (2022). Use of a p64 MW Flow Diverter with Hydrophilic Polymer Coating (HPC) and Prasugrel Single Antiplatelet Therapy for the Treatment of Unruptured Anterior Circulation Aneurysms: Safety Data and Short-term Occlusion Rates. Cardiovasc. Intervent Radiol..

[25] Schwab R., Kabbasch C.h., Goertz L., Kaschner M., Weiss D., Loehr C., Wensing H., Bester M., Simgen A., Kemmling A. (2025). The DERIVSO 2 Heal Embolization Device in the Treatment of Ruptured and Unruptured Intracranial Aneurysms: A Retrospective Multicenter Study. Clin. Neuroradiol..

[26] Weiss D., Vach M., Ivan V., Muhammad S., Hofmann B.B., Neyazi M., Turowski B., Kaschner M. (2025). Comparison of antithrombogenic coated and uncoated flow diverters in ruptured and unruptured cerebral aneurysms. J. Neuroimaging.

[27] Scarcia L., Clarençon F., Dmytriw A.A., Shotar E., Jabbour P., Psychogios M., Sporns P., Puri A.S., Hassan A.E., Algin O. (2025). Silk Vista Baby for the treatment of distal anterior cerebral artery aneurysms. CRETA investigators. Neuroradiology.

[28] Vivanco-Suarez J., Salem M.M., Sioutas G.S., Covell M.M., Jankowitz B.T., Srinivasan V.M., Burkhardt J.-K. (2023). Safety and efficacy of the p48 MW and p64 flow modulation devices: A systematic review and meta-analysis. Neurosurg. Focus..

[29] Piñana C., Remollo S., Zamarro J., Werner M., de Rueda M.E., Vega P., Hernandez D., Murias E., Rivera E., Olier J. (2023). Derivo embolization device for intracranial aneurysms: A Spanish multicenter retrospective study. Neurointerv. Surg..

[30] Fan Y., Lei J., Fei F., Liu J., Liu Y. (2023). A novel flow diverter device (Tubridge) for the treatment of intracranial aneurysms: A systematic review and meta-analysis. Neurosurg. Rev..

